# Transcriptome sequencing and differential gene expression analysis of the schistosome-transmitting snail *Oncomelania hupensis* inhabiting hilly and marshland regions

**DOI:** 10.1038/s41598-017-16084-z

**Published:** 2017-11-17

**Authors:** Jin-Song Zhao, An-Yun Wang, Hua-Bin Zhao, Yan-Hong Chen

**Affiliations:** 1 School of Basic Medicine, Wannan Medical College, Wuhu, 241002 China; 2 School of Public Health, Wannan Medical College, Wuhu, 241002 China; 3 College of Life Sciences, Wuhan University, Wuhan, 430072 China

## Abstract

The freshwater snail *Oncomelania hupensis* is the unique intermediate host of the blood fluke *Schistosoma japonicum*, which is the major cause of schistosomiasis. The snail inhabits two contrasting environments: the hilly and marshland regions. The hilly snails are smaller in size and have the typical smooth shell, whereas the marshland snails are larger and possess the ribbed shell. To reveal the differences in gene expression between the hilly and marshland snails, a total of six snails, three per environment, were individually examined by RNA sequencing technology. All paired-end reads were assembled into contigs from which 34,760 unigenes were predicted. Based on single nucleotide polymorphisms, principal component analysis and neighbor-joining clustering revealed two distinct clusters of hilly and marshland snails. Analysis of expression changes between environments showed that upregulated genes relating to immunity and development were enriched in hilly snails, while those associated with reproduction were over-represented in marshland snails. Eight differentially expressed genes between the two types of snails were validated by qRT-PCR. Our study identified candidate genes that could be targets for future functional studies, and provided a link between expression profiling and ecological adaptation of the snail that may have implications for schistosomiasis control.

## Introduction

The blood fluke *Schistosoma japonicum* (Platyhelminth: Trematoda) occurs in China and, to a lesser extent, in the Philippines and parts of Indonesia, and human infection by the blood fluke causes a major public health problem especially in lake and marshland regions^1,2^. In China, up to 11.6 million people from 12 provinces have been infected by this pathogen since 1949^1–3^. Although over 40 species of mammals were identified to be definitive hosts of the blood fluke^1,4^, only one species of animal is its intermediate host: the freshwater snail *Oncomelania hupensis* (Gastropoda: Pomatiopsidae)^2,3^. Since mid-1950s, concerted control efforts, including molluscicide treatment, biological control and social intervention, have obtained remarkable achievements in decreasing the prevalence of infected people by the blood fluke in China^5,6^. By contrast, the construction of the Three Gorges Super Dam has dramatically changed the natural environmental condition in southern and central China, which would greatly impact the distribution of *O. hupensis* and the transmission of *S. japonicum* ^7,8^. Currently, humans infected by the blood fluke were concentrated along the Yangtze River’s middle and lower reaches and around great lakes in the central China^9^. Because of the intimate connection between the transmission of schistosomiasis and the geographical range of *O. hupensis* ^10,11^, the control of *O. hupensis* remains critical in prevention of schistosomiasis^7,12^.

As an amphibious animal, the schistosome-transmitting snail *O. hupensis* mainly inhabits two contrasting environments: the marshland region and the hilly region^13,14^. The hilly snails have the typical smooth shell and are located in hilly/mountainous regions along the middle and lower reaches of the Yangtze River, while the marshland snails are morphologically characterized by the ribbed shell and distribute in low-lying lake/marshland regions along the middle and lower reaches of the Yangtze River^13,14^. Snails in hilly region possess the smaller size with a height from 5.8 to 6.9 mm, whereas those in marshland regions have the larger size with a height about 7.5 mm and sometimes over 10.0 mm^14^. A total of four subspecies of *O. hupensis* were recognized in China, namely *O. hupensis hupensis*, *O. hupensis robertsoni*, *O. hupensis tangi* and *O. hupensis guangxiensis* ^9,15^. However, the snails occurring along the middle and lower reaches of the Yangtze River belong to the same subspecies *O. hupensis hupensis* ^16^.

Adults of the snail *O. hupensis* frequently occur in fertile soils with luxuriant growth of weeds^14^. Different ecological factors, such as the latitude, humidity, temperature, water level, soil and vegetation, would have critical impacts on the distribution of *O. hupensis*. In particular, water is one of the essential factors for development and reproduction of the snails, which are difficult to survive in dry conditions^17,18^. Indeed, the ratio of days in waterlogging to days in bottomland is a key factor to restrict the density and distribution of *O. hupensis* ^16^. Specifically, the emergence in bottomland for months from February to May permits *O. hupensis* to lay eggs, and the waterlogging after May is required for their egg-hatching and the development of young snails^16^. As another major ecological factor affecting the development of *O. hupensis*, vegetation not only keeps snails warm in winter and supplies shelter from the blazing sunlight in summer, but also provides plenty of nutrients during their life cycles^14^.

Owing to the apparent morphological differences and ecological variations, the snails inhabiting the marshland and hilly regions are expected to respond differentially by increasing or decreasing the expression levels of genes associated with morphological and ecological variations, as shown in other aquatic animals^19–21^. Thus, transcriptome responses to environmental changes in the snail would help understand how they adapt to local environments, which may have important implications for controls against *S. japonicum* infection.

In this study, we used RNA sequencing technology (RNA-seq) to characterize the transcriptome profiling of the snail *O. hupensis* from the two distinct habitats: the hilly region and the marshland region. Our study provided a link between gene expression profiling of the snail and its ecological adaptation, and identified candidate genes that could be targets for future studies.

## Results

### Molecular identification of specimens

Morphological identification of the snails is straightforward, because the hilly snails are smaller (5.8–6.9 mm in height) and have the typical smooth shell, while the marshland snails are larger (7.5–10 mm in height) and have the ribbed shell^13,14^. To validate our morphological diagnosis of specimens, we undertook the molecular identification using the 13 mitochondrial coding sequences. Specifically, we took the 13 mitochondrial genes from each of the four subspecies of *O. hupensis* in China and one subspecies in Philippines (*O. hupensis quadrasi*) downloaded from the GenBank database, and extracted the same genes in the six snails from our transcriptome assemblies. The 13 genes were concatenated and aligned, and the resulting alignment was used to conduct phylogenetic analysis by the maximum likelihood and Bayesian approaches. Both phylogenetic approaches revealed that all hilly snails or all marshland snails formed a monophyletic group, and that all snails examined in this study were clustered with the subspecies *O. hupensis hupensis* rather than other subspecies **(**Supplementary Fig. S1 **)**. Moreover, the phylogenetic relationships among the four subspecies of China recovered in this study were exactly same to that inferred from *16SrRNA* sequences^22^. Thus, our molecular evidence unambiguously suggests that the six snails studied here belong to the same single subspecies *O. hupensis hupensis*.

### RNA-seq de novo assembly

**Figure 1:**
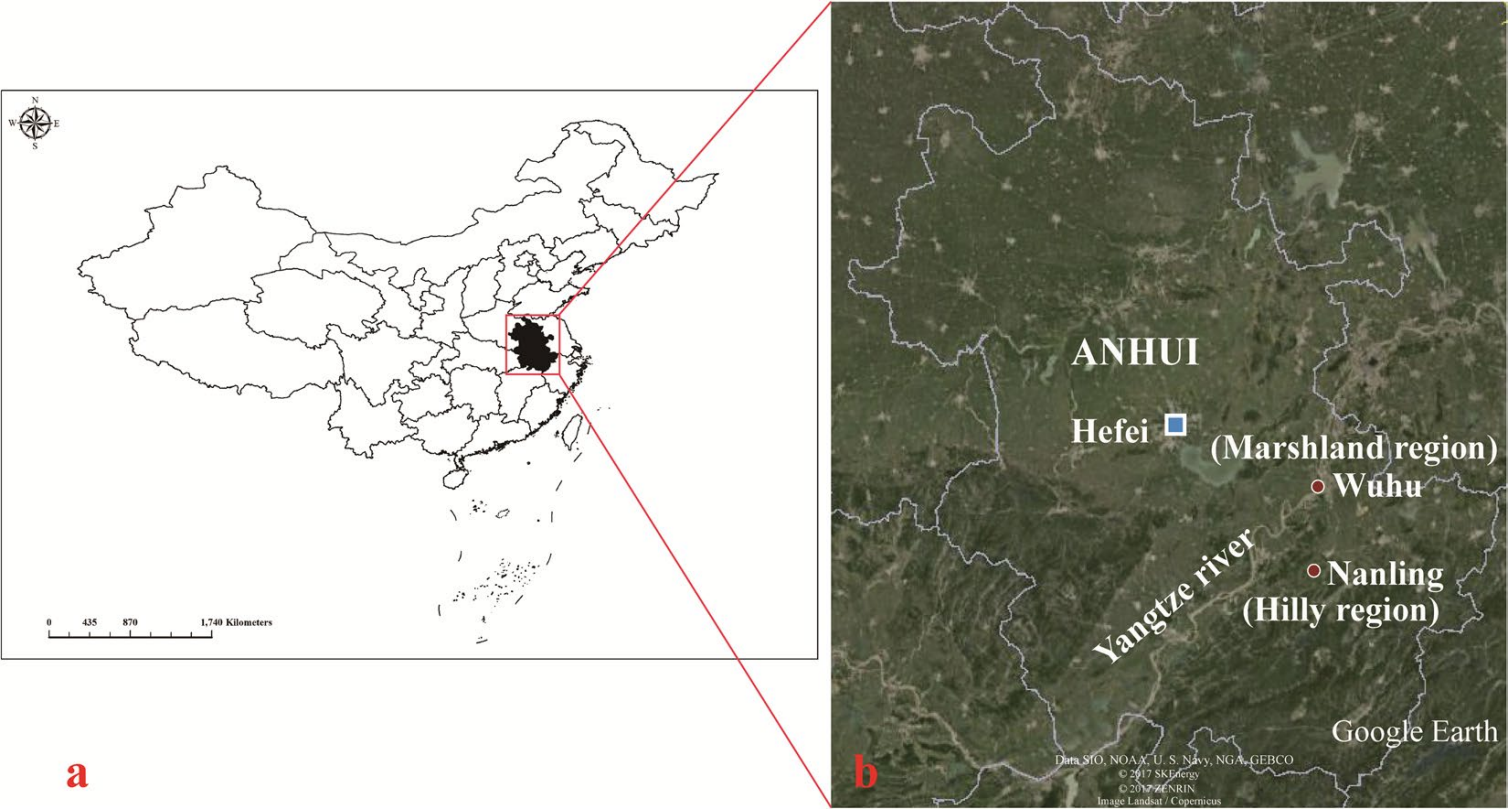
Geographical locations of the sampling regions. (**a**) Location of Anhui province in China; The map was generated using ArcGIS version 10.2 (ESRI, Redlands, CA, USA); (**b**) Sampling sites in Wuhu City and Nanling County within Anhui province. The satellite images were taken from Google Earth http://earth.google.com/.

The total number of singleton reads from all six individuals of the snail *O. hupensis* (three from hilly region and three from marshland region) **(**Fig. 1 **)** was 258,878,390, with the number of singleton reads from each individual ranging from 38 to 51 million (M) (Table 1). After trimming, a total number of 3,735,164 reads with the length less than 25 base pair (bp) were discarded from all samples and the discarded reads for each sample was concentrated on a small percentage (1.3-1.56%). More than 98.4% of total reads from each sample were retained for de novo transcriptome assembly (Table 1). The de novo transcriptome assembly was generated by all retained reads from the six samples. The assembly contained a total of 564,625 contigs with an average length of 553 bp and an N50 value of 667 bp **(**Table 2 **)**. After removing the redundancies, we retained contigs with an FPKM (fragments per kilobase per million fragments mapped) value no less than two in at least two samples from either habitat. To avoid the mapping bias caused by incomplete fragements, we filtered the shorter fragmented contigs that were annotated by the same proteins, and retained the longest contigs. As such, we derived 34,760 unigenes (i.e. unique putative genes) from these contigs **(**Table 2 **)**. All unigenes have an N50 value of 1,243 bp with a mean length of 711 bp (Table 2). To visualize global transcriptomic patterns, we undertook hierarchical clustering of expression levels for all unigenes **(**Supplementary Fig. S2 **)**. It appears that all hilly samples formed a cluster while all marshland samples formed another **(**Supplementary Fig. S2 **)**. Raw transcriptome data of the six snails are available from the NCBI Sequence Read Archive under the accession number SRP103982.

**Table 1:** Details on the raw data and quality-filtered RNA-seq data for *Oncomelania hupensis*. Reads that were < 25 base pairs after quality trimming were discarded from use in the transcriptome assembly.

**Sample**	**Paired-end reads**	**Total singleton reads**	**Discarded**	**Percentage discarded**	**Retained**	**Percentageretained**
H1	25,716,346	51,432,692	668,936	1.30	50,763,756	98.70
H2	22,804,257	45,608,514	626,786	1.37	44,981,728	98.63
H3	21,610,090	43,220,180	675,010	1.56	42,545,170	98.44
M1	19,323,214	38,646,428	573,672	1.48	38,072,756	98.52
M2	20,050,813	40,101,626	591,952	1.48	39,509,674	98.52
M3	19,934,475	39,868,950	598,808	1.50	39,270,142	98.50

**Table 2:** Details of the snail (*O. hupensis*) transcriptome assembly. Contigs refer to continuous lengths; nt = nucleotides.

Assembly metric	*Oncomelania hupensis*
**Contigs**	
Number	564,625
Mean length (nt)	553
N50 statistics (nt)	667
**Unigenes**	
Number	34,760
Mean length (nt)	711
N50 statistics (nt)	1,243

### Functional annotation of the transcriptome

**Figure 2:**
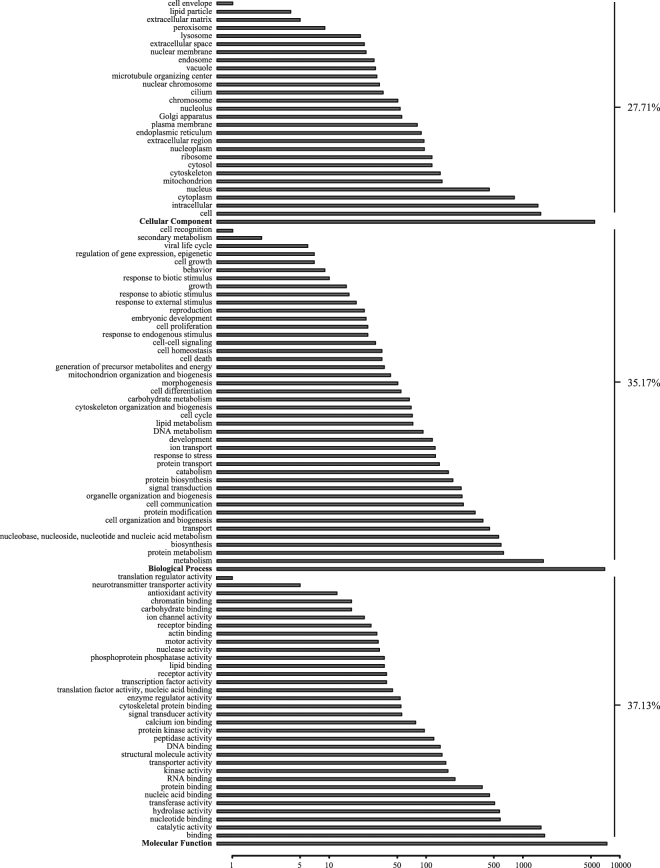
The gene ontology categories in the transcriptome of *Oncomelania hupensis*. Each of three major categories was shown in bold, while all subcategories within each major category were shown in plain on the y-axis. The bar length represents the log10 transformation of unigene number, and the actual number of unigenes is shown on the x-axis. The percentage of unigene counts is presented on the right.

Of all unigenes, a total of 8,584 (24.69%) or 6,080 (17.49%) have a significant BLAST hit when annotated against NR (non-redundant) or Swiss-prot database. For GO (gene ontology) classification, NR annotated unigenes were converted to produce 3,262 GO terms via Blast2Go program^23^. Of the remaining 26,176 unigenes that are unannotated, 4,823 harbored a predicted open reading frame (ORF) with a length longer than 150 nucleotides. Through annotating these ORFs with the InterPro protein signature databases, we found 175 unigenes that were assigned to 82 GO terms. After removing the replicated terms generated by the Blast2Go program^23^ and InterProScan^24^, additional 16 GO terms were obtained. All these GO terms were categorized into 106 ancestral classes, and were depicted in detail (Fig. 2). Molecular functions were predominant at 37.13%, with the major categories encompassing “binding”, “catalytic activity”, “hydrolase activity” and “nucleotide binding”. Biological processes accounted for 35.17%, with “metabolism”, “protein metabolism”, “biosynthesis” and “nucleobase, nucleoside, nucleotide and nucleic acid metabolism” most presented. Cellular components possessed the lowest proportion at 27.71%, and mainly included “cell”, “intracellular”, “cytoplasm” and “nucleus” (Fig. 2).

### SNP calling and population clustering

**Figure 3:**
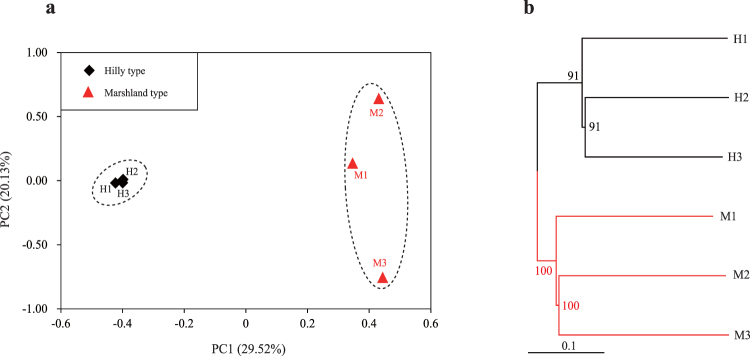
Population clustering analysis. (**a**) Principal component analysis (PCA) of the snails. Black squares (H1-H3) refer to three individuals of hilly snails; Red triangles (M1–M3) indicate three marshland snails. (**b**) Neighbor-joining (NJ) tree based on all single nucleotide polymorphisms (SNPs). Black, hilly snails; Red, marshland snails.

A total of 326,685 single nucleotide polymorphisms (SNPs) were identified from the six samples. Two distinct population clusters, the marshland type and the hilly type, were recognized by the principal component analysis (PCA) (Fig. 3a) and the neighbor-joining (NJ) tree (Fig. 3b) based on all SNPs. In the PCA, the first principal component explained 29.52% while the second principal component explained 20.13% of the genetic differences (Fig. 3a). In the NJ tree, the two populations were completely separated (Fig. 3b).

For comparison, we generated a Multi-Dimension Scale (MDS) plot that shows the expression divergence of snails inferred from RNA-seq read counts. Consistent with the PCA plot inferred from SNPs (Fig. 3a), the MDS plot also revealed two distinct clusters of hilly and marshland snails **(**Supplementary Fig. S3 **)**. In contrast to the PCA analysis showing lower genetic divergence in hilly snails and higher divergence in marshland ones (Fig. 3a), the MDS analysis identified higher expression divergence within hilly snails and lower divergence within marshland ones **(**Supplementary Fig. S3 **)**. This disparity suggests that coding-sequence divergence and expression divergence are not coupled in the snails.

### Differential gene expression between two habitat types of snails

**Figure 4:**
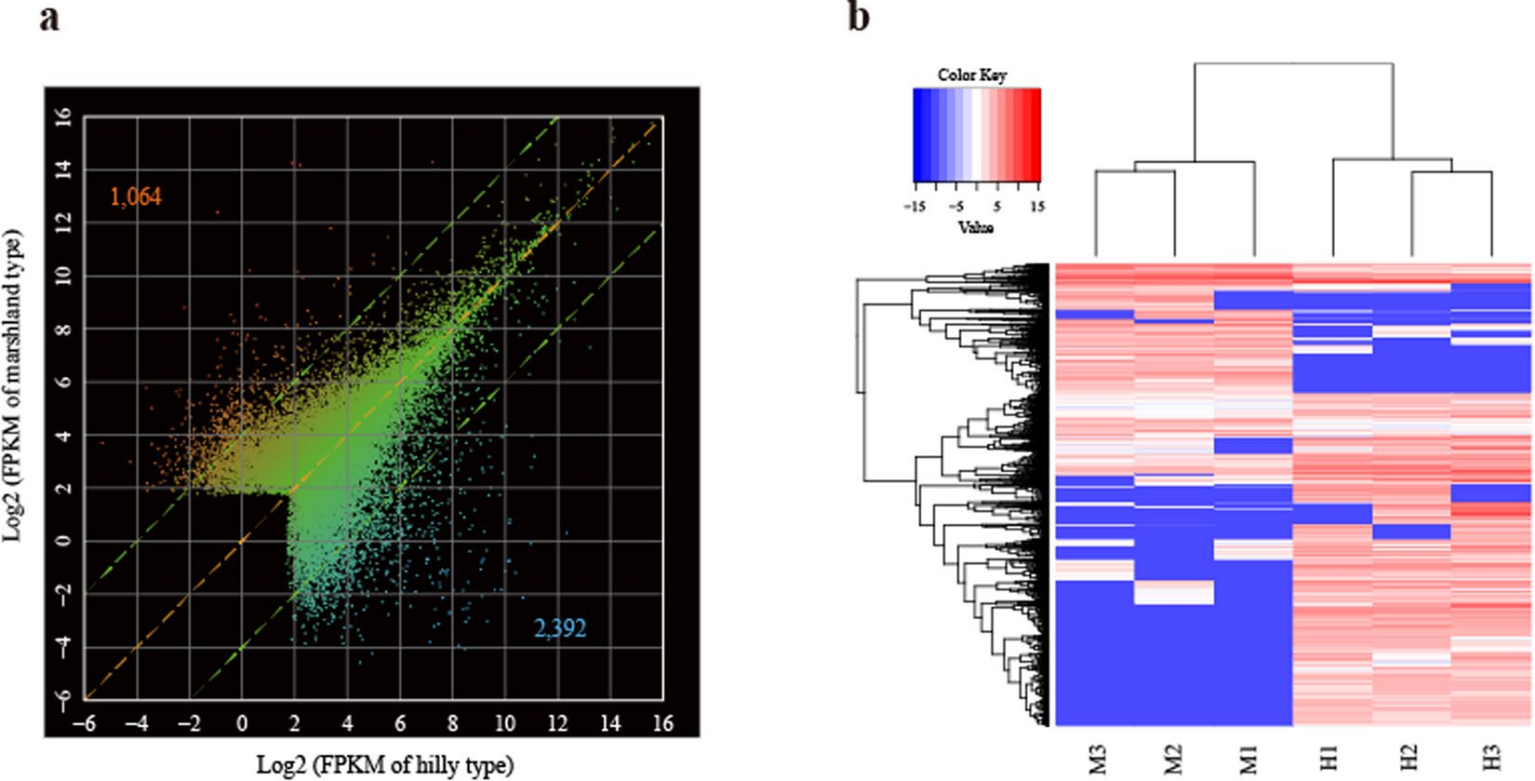
Analysis of differentially expressed unigenes. (**a**) Comparison between the hilly and marshland snails via RNA-seq measured in fragments per kilobase per million fragments mapped (FPKM). Each dot represents a unigene, and two green lines bracket unigenes having less than 4-fold differences in expression between environments; and the unigenes with ≥ 4-fold differences in expression were considered to be differentially expressed in this study. A total of 1,064 genes were upwardly expressed and 2,392 were downwardly expressed in hilly snails relative to marshland snails; (b) Heatmap of all 3,456 differentially expressed unigenes between the hilly and marshland snails.

A total of 3,456 unigenes were differentially expressed in the snails from the marshland region when compared with those from the hilly region, making up 9.94% of the total 34,760 unigenes. The numbers of upwardly expressed and downwardly expressed unigenes in the marshland and hilly snails were 1,064 and 2,392, respectively (Fig. 4a), and all differentially expressed genes were visualized by the hierarchical clustering of expression levels (Fig. 4b). Among all differentially expressed unigenes, 121 in hilly snails and 111 in marshland snails could be successfully annotated by the Swiss-prot database, respectively.

For the upwardly expressed genes in the marshland snails, 65 GO terms were significantly enriched with a *p*-value less than 0.05 (Supplementary Table S1). Specifically, 42 GO terms such as “translation”, “peptide biosynthetic process”, “ribosome assembly” and “organonitrogen compound metabolic process” were over-represented in the “biological process” category; 11 GO terms including “structural constituent of ribosome”, “structural molecule activity”, “rRNA binding” and “translation elongation factor activity” were over-represented in the “molecular function” category (Supplementary Table S1). For the upwardly expressed genes in the hilly snails, a total of 70 GO terms were significantly enriched with a *p*-value less than 0.05 (Supplementary Table S2). In the “biological process” category, 36 GO terms including “immune system process”, “protein metabolic process”, and “development” were over-represented. The GO terms such as “structural constituent of ribosome”, “rRNA binding”, and “structural molecule activity” were over-represented under the “molecular function” category (Supplementary Table S2).

**Figure 5:**
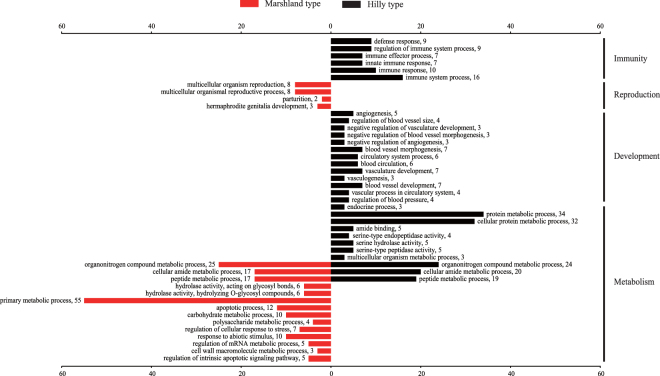
Gene ontology (GO) enrichment analysis of differentially expressed unigenes between the hilly and marshland snails. Gene number is provided adjacent to each GO term.

Functional enrichment analyses of GO terms for differentially expressed genes were compared between the two habitats (Fig. 5). The analysis of differentially expressed genes indicated that a large number of GO terms associated with metabolic processes and enzyme activities were enriched in snails from both habitats (Fig. 5). Furthermore, several GO terms related to larval development and immunity were significantly enriched in hilly snails (Fig. 5). By contrast, only four GO terms relating to reproduction were enriched in marshland snails (Fig. 5). The highly expressed unigenes in each habitat that could be annotated by the Swiss-prot database were listed **(**Table 3 **)**. Most genes that are highly expressed in each habitat were involved in metabolic activities (Table 3). Notably, nine genes associated with reproduction were significantly upregulated in marshland snails (Table 3). By contrast, in hilly snails, 18 and 14 upregulated genes were related to immunity and development, respectively (Table 3).

**Table 3:** Summary of gene ontology (GO) enrichment in highly expressed genes in the hilly or marshland snails. Of note, the listed genes are only a part of differentially expressed unigenes that were successfully annotated by the Swiss-prot database; eight genes examined by qRT-PCR are shown in bold.

Function	Hilly type		Marshland type	
	enrichment terms	Major genes	enrichment terms	Major genes
Immunity	GO:0002252, GO:0002376, GO:0002682, GO:0006952, GO:0006955, GO:0045087	*ACE, ALPK1, BIRC3, CD180, CHIA, CP3AT, CRIP1, DOPO, FCER2, GGT1, LRP5, PARPT, RS14, RS30, SAMH1, SFTPD, TIE2, VNN1*	None	None
Reproduction	None	None	GO:0007567, GO:0032504, GO:0040035, GO:0048609	*ACR, CP1A1, DNJA1, EF2, HSP7C, PGDH, R23A2, RL11,YBOX 3*
Development	GO:0001525, GO:0001568, GO:0001570, GO:0001944, GO:0003013, GO:0003018, GO:0008015, GO:0008217, GO:0016525, GO:0048514, GO:0050880, GO:1901343, GO:2000181	*ACE, ANPRA, CP2J5, DOPO,* ***FAP, LRP5*** *, NOTC3, PARPT, PTPRM, RHOA, SEPR, SYWM, TEN-1, TIE2*	None	None
Metabolism	GO:0004252, GO:0006518, GO:0008236, GO:0017171, GO:0019538, GO:0033218, GO:0043603, GO:0044236, GO:0044267, GO:0050886, GO:1901564	*ACE, ACRO, ALPK1, ANPRA, BIRC3, CO6A5, CP2J5, CRHBP, CRIP1, DNER, DOPO, EF1G, EIF3A, GGT1, GRM4*, ***LRP5*** *, NEK4, NOTC3*, ***P4HA2*** *, PARPT, PPIA, PPSA, PRS41, PTPRM, RFIP1, RHOA, RL222, RL23, RL5A, RL6, RL7, RL7A, RLA22, RS13, RS14, RS15A, RS26B, RS30, RS5A, RS9, SAMH1, SEPR, ST38L, SYWM, TDH, TIE2, TRY1, YBX2B*	GO:0004553, GO:0005975, GO:0005976, GO:0006518, GO:0006915, GO:0009628, GO:0016798, GO:0043603, GO:0044036, GO:0044238, GO:0080135, GO:1901564, GO:1903311, GO:2001242	*1433E, ADT, AMY2B, ASL1* ***, AS3MT, ATHL1*** *, ATS17, ATS18*, ***BHMT*** *, BI1, BPL1, CCAR2, CHI1, CHI10, CHSTE, CP1A1, DBP2, DDX11, DNJA1, DRAM2, ECHP, EF1A1, EF2, ERG7, FUBP2, GALM, GATM, GUN6, HSP60, HSP7C, ING4, KAD4, LRWD1, MATK, MPU1*, ***PABP4*** *, PCKGC, PDIA1, PEAM3, PGDH, PTBP3, R23A2, RBP1, RL11, RL4B, RL5, RL7, RL8, RLA2, RP12, RS15, RS18, RS32, RS8, SM3L3, TEP1, TRX1, TYPH, WOS2, YBOX 3*

### Validation of RNA-seq data by quantitative real-time PCR (qRT-PCR)

**Figure 6:**
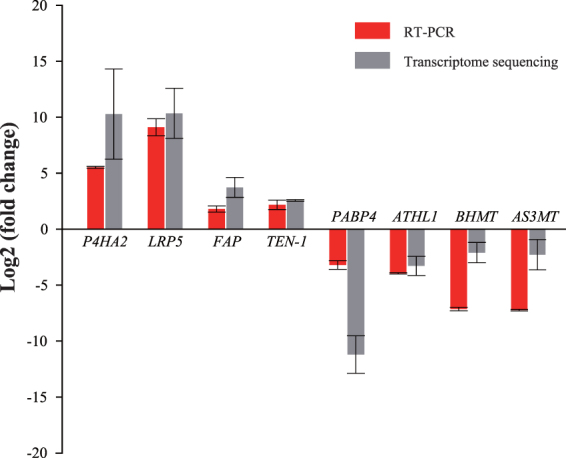
Expression profiles of eight differentially expressed genes. Expression levels in marshland snails were calculated as the control for each gene. Red bars represent the relative expression levels in hilly snails by RT-PCRs and grey bars indicate the relative expression levels in hilly snails by transcriptome sequencing. All relative expression values were shown with log2 (fold change) on the Y axis.

To verify the expression pattern shown by RNA-seq data, a total of eight differentially expressed genes between the two types of snails were selected for validation using qRT-PCR. Among these genes, four (*LRP5*, *FAP*, *TEN-1*, and *P4HA2*) were found to be highly expressed in hilly snails whereas the other four (*AS3MT*, *ATHL1*, *BHMT*, and *PABP4*) were identified to be upregulated in marshland ones by RNA-seq data **(**Table 3 **)**. Functionally, one gene (*LRP5*) is involved in immunity, three genes (*FAP*, *LRP5*, and *TEN-1*) are asscociated with development, and five genes (*AS3MT*, *ATHL1*, *BHMT*, *LRP5*, *P4HA2*, and *PABP4*) are related to metabolism **(**Table 3 **)**. Our qRT-PCR measurements for the eight genes revealed similar trends of expression changes estimated from the RNA-seq data (Fig. 6).

## Discussion

This study characterized the de novo transcriptome assembly of the snail *O. hupensis*. GO term classification indicated that many unigenes are indispensable in metabolism and catalytic activities. SNP calling and population clustering suggested that genetic variation between the two habitat types within a relatively smaller geographical area was higher than that within population, which implied that habitat type might play a vital role in shaping the genetic differentiation of *O. hupensis*. Analysis of expression profiles showed that the majority of differentially expressed genes were related to metabolism. In addition, our study also revealed that upregulated genes involving in reproduction were enriched in marshland snails, whereas those genes relating to immunity and development were over-represented in hilly snails.

The RNA-seq generated high-quality reads for de novo transcriptome assembly of *O. hupensis*. For each contig cluster, the isoform with the highest expression level, measured by FPKM values, was selected as a unigene. We next filtered the shorter fragmented contigs that were annotated by the same proteins, and retained the longest contigs. The obtained contigs were selected as unigenes for subsequent analyses. Only 8,584 and 6,080 of the total 34,760 unigenes were annotated by the NR database and Swiss-prot database, accounting for 24.69% and 17.49%, respectively. The number of annotated unigenes was considerably limited, possibly because functions of many unigenes are usually specific to a certain lineage or a particular environment^25^. Furthermore, there were only five species in Gastropoda that had draft genome sequences. Specifically, two of the five species belong to the order Basommatophora, namely *Lymnaea stagnalis* ^26^ and *Biomphalaria glabrata* ^27^. The remaining three species *Conus tribblei* ^28^, *Aplysia californica* ^29^ and *Lottia gigantea* ^30^ belong to the orders Neogastropoda, Anaspidea, Patellogastropoda, respectively. No genome data for Pomatiopsidae had been published thus far, which may also limit the number of annotated unigenes. “Molecular function” made up the majority of GO categories that the unigenes in *O. hupensis* fell into, followed by “biological process” and “cellular component” (Fig. 2). While in another gastropod mollusc, the wandering snail *Radix balthica*, “biological process” represented the largest category; “cellular component” is the second largest category, and “molecular function” occupied the smallest proportion of unigenes^31^.

The transcriptome GO terms of *O. hupensis* were partially overlapped with those of another snail *Radix balthica* and several species of crustaceans^31–35^. Specifically, within the category “molecular function”, the subcategories “catalytic activity” and “binding” with the most mapped transcripts were also identified in *R. balthica* ^28^ and some crustaceans^32–35^. Under the category “biological process”, two subcategories “metabolism” and “cell organization and biogenesis” with a large amount of mapped transcripts were also observed in *R. balthica* ^28^. However, the subcategory “biological regulation” with a great many mapped transcripts in *R. balthica* ^28^, *Oncopeltus fasciatus* ^30^ and *Benista tabaci* ^35^ was not present in *O. hupensis* (Fig. 2). Of note, we cannot rule out that differences observed between *O. hupensis* and other species in transcriptome GO terms may have resulted from the small number of annotated unigenes derived from the transcriptome assembly.

The high-quality paired-end reads from the six snails were mapped to the reference transcriptome to identify SNPs. SNPs were then compared among samples and used to conduct the phylogenetic reconstruction and principal component analysis. According to the principal component analysis, the individuals from the same environment were clustered, implying that genetic difference between the two habitat types was relatively higher than that within one habitat type. Indeed, populations of *O. hupensis* from different areas had different degrees of genetic variation^36^. Likewise, *O. hupensis* from various habitats had an obviously different susceptibility against *S. japonicum* ^37–40^. Our study also indicated that the three samples from the marshland region harbored higher genetic difference than those from the hilly regions, which was consistent with earlier genetic evidence^16,41^. For the hilly snails, our principal component analysis showed that the hilly individuals formed a compact group **(**Fig. 3a **)**, which indicated that there was a considerable degree of sequence similarity among the hilly snails. However, the bootstrap values within the hilly snails were lower compared with those in the marshland snails, which were 100% at each node **(**Fig. 3b **)**. This observation suggests that in spite of the higher sequence similarity within the hilly snails, the number of phylogenetically informative sites in hilly snails may be smaller than that in marshland snails.

Because ecological factors, such as water level, humidity, temperature, soil and vegetation, were significantly different between the hilly and marshland environments^42^, regulation in gene expression can be the result of animals reacting to the environmental stresses^43,44^. Upwardly expressed genes in hilly snails are mainly involved in several physiological activities, including metabolism, immunity and development. Upwardly expressed genes in marshland snails are mostly concentrated on terms relating to metabolism and reproduction. A large amount of highly expressed genes in both types of the snails were enriched in GO terms relating to metabolism **(**Fig. 5 **)**. Four enriched GO terms, including “cellular amide metabolic process”, “organonitrogen compround metabolic process” and “peptide metabolic process”, are shared in both types of the snails **(**Fig. 5 **)**, which demonstrated that many genes were participated in macromolecule metabolic processes. Furthermore, several GO terms such as “carbohydrate metabolic process” and “polysaccharide metabolic process” were enriched in marshland snails, whereas GO terms such as “serine hydrolase activity” and “endocrine process” were over-represented in hilly snails **(**Fig. 5 **)**. The marshland snails harbor more highly expressed genes associating with metabolism than the hilly snails **(**Fig. 5 **)**, possibly because the larger snails in size from the marshland region tend to have a higher basal metabolic rate^45^.

A total of 18 genes related to immunity were highly expressed in hilly snails (Table 3). Previous studies revealed that snails from different habitats had a significant difference in the susceptibility against *S. japonicum* ^39,40^. For the subspecies *O. hupensis hupensis*, the marshland snails harbored a higher infection rate to the local *S. japonicum* than the hilly snails^42^. For *CHIA* gene, as a member of an atypical glycoside hydrolase family 18, were proved to possess a chitin-binding Peritrophin-A domain to bind and break down chitin, but also play a role in host defense against fungi, bacteria, and other pathogens in many species^46–48^. The tissue-specific expression of chitinase in triangle snail mussel *Hyriopsis cumingii* suggested that a chitinase-3 gene were highly expressed after shell damage^49^. In *Crassostrea gigas*, two chitinase-like proteins (CLPs) were transcriptionally stimulated in haemocytes when confronted with bacterial lipopolysaccharide challenge, also suggested that these two CLPs may play a role in immunity^50^.

Nine genes related to reproduction were found to be highly expressed in the marshland snails **(**Table 3 **)**. Since *O. hupensis* is dioecious and has an oviparous reproductive system, various environmental factors may play vital roles in mating and egg-laying^42^. For example, highly expressed gene *ACR* is a typical serine proteinase present in acrosome of mature spermatozoa and play a vital role in penetrating the zona pellucida of the ovum^51^. Highly expressed *ACR* may help the marshland snails maintain a relative higher fertilization rate than the hilly snails.

A total of 14 upregulated genes had been enriched in 13 GO terms associated with organismic development in hilly snails **(**Table 3 **)**. For example, *LRP5*, encoding the low-density lipoprotein receptor-related protein 5, plays an essential role in canonical Wnt pathway and skeletal homeostasis^52^. Several studies had indicated that *LRP5* in mice and humans controls bone formation by inhibiting the expression of TPH1 (Tryptophan hydroxylase 1), a rate-limiting biosynthetic enzyme for serotonin in enterochromaffin cells of the duodenum^53–56^. Another highly expressed gene, *NOTC3*, was found to inhibit the osteoblast differentiation^57,58^, and also function in prenatal skeletal development and postnatal bone remodeling^58^. Considering the smaller size and thinner shell in hilly snails when compared with the rib-shelled marshland snails^59^, upregulated genes such as *LRP5* and *NOTC3* might play a role in the shell formation. This hypothesis awaits experimental validation in future.

Taken together, we characterized the differences in gene expression of the schistosome-transmitting snail *O. hupensis* inhabiting the hilly and marshland environments by RNA sequencing technology. The majority of differentially expressed genes between environments were involved in metabolism, and upregulated genes relating to development and immunity were enriched in hilly snails, while those associated with reproduction were over-represented in marshland snails. Our study identified candidate genes that could be targets for future functional studies, and provided a link between expression profiling and ecological adaptation of the snail that may have implications for schistosomiasis control.

## Materials and Methods

### Sample preparation and RNA isolation

Wild uninfected adults of *Oncomelania hupensis* used in this study were collected in Anhui Province, southeast China in October 2015. Samples were obtained from the Yangtze River marshland region in Wuhu city (geographical coordinates: 31°20′N, 118°21′E) and the hilly region in Nanling county (30°48′N, 118°13′E), respectively **(**Fig. 1 **)**. For each habitat, three individuals of the snails were used as three biological replicates in transcriptome profiling. We examined microscopically in the laboratory to check infection in each individual of snails, and those without helminthic infection were used for RNA isolation. Following the manufacture’s protocol, total RNAs were isolated using Trizol (Invitrogen, CA, USA) and were subsequently quantified by a NanoDrop spectrophotometer (NanoDrop Technologies, DE, USA).

### Library preparation and RNA sequencing

Six snails were individually used to construct RNA-seq libraries using the Illumina Truseq RNA Sample Preparation Kit (San Diego, CA, USA), and then quantified by Qubit (Life Technologies) accordingly to the manufacturer’s protocol. Details of library preparation for RNA-seq were described elsewhere^60,61^. Six paired-end libraries with an insert size of approximately 200 bp were sequenced to generate 125 bp paired-end reads on the Illumina HiSeq. 2500 sequencing platform.

### Molecular identification of specimens

To clarify the phylogenetic relationships among the six snails and determine which subspecies these samples belong to, the de novo transcriptome assembly was conducted separately for each snail. All raw sequence reads were assessed by FastQC version 10.1 (www.bioinformatics.bbsrc.ac.uk/projects/fastqc) and were processed using Trimmomatic version 0.32^62^ to remove the residual adaptors and low quality sequences as well as ambiguous sequences. Trimming parameter settings were detailed as follows: we removed a total of 13 bp from the start of sequence reads, with a Phred quality score of leading and trailing bases < 4, and performed a sliding window approach once the average Phred quality score dropped below 15, and discarded those sequence reads < 25 bp. Six transcriptome assemblies were processed by the program Trinity version 2.2.0^63^ with default parameters.

TBLASTN searches^64^ were applied to identify the 13 mitochondrial protein coding genes from each of the six transcriptome assemblies. The same genes of four subspecies in China and one subspecies in Philippines were downloaded from the GenBank database (http://www.ncbi.nlm.nih.gov/genbank) under accession numbers as follows: *O. hupensis hupensis*, JF284687; *O. hupensis tangi*, JF284695; *O. hupensis guangxiensis*, JF284696; *O. hupensis robertsoni*, JF284697 and *O. hupensis phillipine*, JF284698. The 13 genes were concatenated and aligned by PRANK version 100802^65^ and poorly aligned position and gaps were removed by GBLOCKS version 0.91b^66^. According to the Akaike information criterion (AIC) and Bayesian information criterion (BIC)^67^, we ran the program jModelTest version 2.1.10^68^ to select the best-fit model for concatenated mitochondrial sequences. PhyML version 3.7^69^ were used to reconstruct for the ML tree under the recommended GTR model with 100 replicates, and MrBayes version 3.2.6^70^ were used to reconstruct the Bayesian tree under the GTR + I + G model with 300 million generation.

### De novo transcriptome assembly and unigene annotation

Paired-end reads from all six libraries were pooled and a de novo transcriptome assembly was conducted by Trinity version 2.2.0 program^63^ with default parameters, and generated a considerable amount of contigs. In order to reduce the effects of erroneous contigs, we separately mapped all filtered paired-end reads from each sample to these contigs by using software RSEM version 1.1.21^71^. We next defined a minimum expression filter of two fragments per kilobase per million fragments mapped (FPKM), and retained contigs with FPKM greater than two in at least two samples from either habitat. For contigs annotated as the same proteins, we removed the shorter fragmented transcripts and retained the longest contigs. The obtained contigs were chosen as a reference unigene set and were remapped by all filtered paired-ended reads from each sample to quantify gene expression level. The FPKM heatmap of all unigenes from six individuals was generated using the gplots package^72^.

The transcriptome-derived unigene set was annotated against NR (NCBI non-redundant) and Swiss-prot databases via BLASTX^64^, with an *e*-value threshold of 1e-5. GO (gene ontology) annotation was performed by the Blast2GO program^23^ with the NR annotation results in XML format as the input file. For those unannotated unigene, the perl script TransDecoder in the Trinity program package was conducted to extract the predicted open reading frame (ORF) at a minimum peptide length threshold of 50. With ORFs as inputs, the program InterProScan version 4.8^24^ was applied to search the InterPro protein signature databases and predict the possible GO terms. GO terms generated by Blast2Go^23^ and InterProScan^24^ were grouped and categorized into three main biological terms, namely “cellular component”, “molecular function” and “biological process”, using a web-based program CateGOrizer with the GO-SLIM classification method^73^.

### SNP calling and population structure analysis

Single nucleotide polymorphisms (SNPs) were identified from filtered paired-end sequence reads by mapping each sample to the final transcriptome-derived unigene set. The mapping was undertaken using the program Burrows-Wheeler Aligner (BWA) version 0.7.8^74^ with the “mem” method and default parameters, after the alignments were converted to the SAM format. The program SAMtools version 1.3.1^75^ with the “view” method was used to convert the SAM format to the BAM format for downstream analyses, with removal of the potential PCR duplicates using the “rmdup” option. All alignment files in BAM format were sorted with the “sort” option, and then grouped together to identify SNPs via the “mpileup” option in SAMtools, with parameters set as “-q 20 -Q 20 -C 50 -t DP SP -m 2 -F 0.002”. The likelihood of each possible genotype was calculated and stored in BCF format via SAMtools. All retained variant sites were filtered by scripts in the BCFtools package^75^, with parameters set as “-d 20 -D 140 -w 5”, in order to acquire variant sites in higher quality. Subsequently, SNPs were captured through the program VCFtools version 0.1.15 package^75^ with the “-remove-indels” option.

Population clustering analysis was conducted using two approaches. The first approach was performed to analyze the population structure based on neighbor-joining (NJ) method. The program TreeBeST version 1.9.2 (sourceforge.net/projects/treesoft/files/treebest) was used to constructed the NJ phylogenetic tree with 1,000 bootstraps, based on concatenated SNPs from all samples. The other approach was based on nonparametric principal component analysis (PCA)^76^. SNPs in the binary PED format were generated via VCFtools with the “-remove-indels-plink” option, and were next used for further analysis via the program PLINK version 1.07 (pngu.mgh.harvard.edu/purcell/plink/)^77^. PCA was conducted using the program Genome-wide Complex Trait Analysis (GCTA) version 1.25.2^78^, with the “-pca 2” option.

In comparison with the population clustering inferred from genetic divergence, we also generated a Multi-Dimension Scale (MDS) plot that shows the expression divergence of snails inferred from RNA-seq read counts. Using the MDS function in edgeR^79^, a multi-dimensional scaling plot of six snails was generated based on contig counts, with the cutoff of one count per million (CPM) at 4.

### Differential gene expression analysis

The filtered paired-end reads from the six samples were separately aligned back to the final transcriptome-derived unigene set with RSEM version 1.1.21^71^. Gene expression was quantified as the total number of fragment counts that uniquely mapped to the unigene set. By using the matrix of fragment counts from each sample, we conducted differential expression analysis via the edgeR program^79^, which is a Bioconductor software package^80^ performed in R environment. The edgeR program could calculate whether there are significant differences in gene expression between marshland and hilly snails, and differentially expressed transcripts resulting from pairwise comparisons were extracted and clustered via TMM (trimmed means of *M* values across samples) normalized FPKM values by a suite of scripts in Trinity package. Differentially expressed genes (DEGs) between the two environments were identified at a significance level of 0.05, with a false discovery rate (FDR) corrected method^81^. Meanwhile, the fold change between the two environments was no less than 4, namely an absolute minimum value of log2-transformed fold change (logFC) equaled to 2.

Along with the selection of differentially expressed genes, an FPKM heatmap was generated via the gplots program^72^ performed in R environment. Differentially expressed genes were annotated against the NR/Swiss-prot database using BLASTX. Functional enrichment tests of these unigenes were analyzed using the DAVID bioinformatics resources^82^. Benjamini-Hochberg multiple testing correction^81^ was used to adjust the significance of functionnal enrichment.

### Validation by quantitative real-time PCR (qRT-PCR)

A total of eight differentially expressed genes between the two types of snails were selected for validation using qRT-PCR. Among these genes, four (*LRP5*, *FAP*, *TEN-1*, and *P4HA2*) were found to be highly expressed in hilly snails whereas the other four (*AS3MT*, *ATHL1*, *BHMT*, and *PABP4*) were identified to be upregulated in marshland ones by RNA-seq data **(**Table 3 **)**. All selected genes have significant BLAST hits against the homologs in NR and Swiss-prot database and have multiple exons for designing primers crossing exon-intron junctions, aiming to eliminate the impact of DNA interference. RNA samples from each individual were the same as those prepared for cDNA library construction. Equal amounts of mRNA were used to generate the first-strand cDNAs by the reverse transcriptase polymerase chain reactions (M-MLV Reverse Transcriptase; Thermo Fisher Scientific, Wilmington, DE). The SYBR Green real-time PCR reaction was executed using the Bio-rad CFX96^TM^ system (Bio-Rad, USA), with the protocol as follows: 95 °C for 5 min followed by 40 cycles of 95 °C for 10 s and 60 °C for 30 s. The gene *β-actin* in *O. hupensis* was also parallelly amplified as the internal control for normalization, and primers of *β-actin* were taken from Zhang *et al.* ^83^. Real-time PCR reactions for each gene from six snails were run in triplicate and each reaction was repeated three times, then the standard curves were achieved. The expression levels of each gene from either habitat snails were normalized to β-actin. The fold-changes were calculated through comparing the hilly snails to the marshland snails using the 2^−ΔΔCt^ method^84^.

### Data availability

Raw RNA-seq data have been deposited to the NCBI Sequence Read Archive under the accession number SRP103982.

## Electronic supplementary material

Supplementary Information
