# Supplementary material for: Transcriptome sequencing and differential gene expression analysis of the schistosome-transmitting snail Oncomelania hupensis inhabiting hilly and marshland regions
Source: Sci Rep. 2017 Nov 17;7:15809. doi: 10.1038/s41598-017-16084-z (PMC5693929; doi:10.1038/s41598-017-16084-z)
Supplement: Supplementary file 1 — Supplementary Information [file 41598_2017_16084_MOESM1_ESM.doc]

Supplementary Information for

**Transcriptome sequencing and differential gene expression analysis of schistosome-transmitting snail *Oncomelania hupensis* inhabiting hilly and marshland regions**

Jin-Song Zhao^1^, An-Yun Wang^2^, Hua-Bin Zhao^3^, and Yan-Hong Chen^3,*^

^1^School of Basic Medicine, Wannan Medical College, Wuhu 241002, China

^2^School of Public Health, Wannan Medical College, Wuhu 241002, China

^3^College of Life Sciences, Wuhan University, Wuhan 430072, China

This document includes:

3 figures: Figure S1 – Figure S3.

3 tables: Table S1 – Table S3.


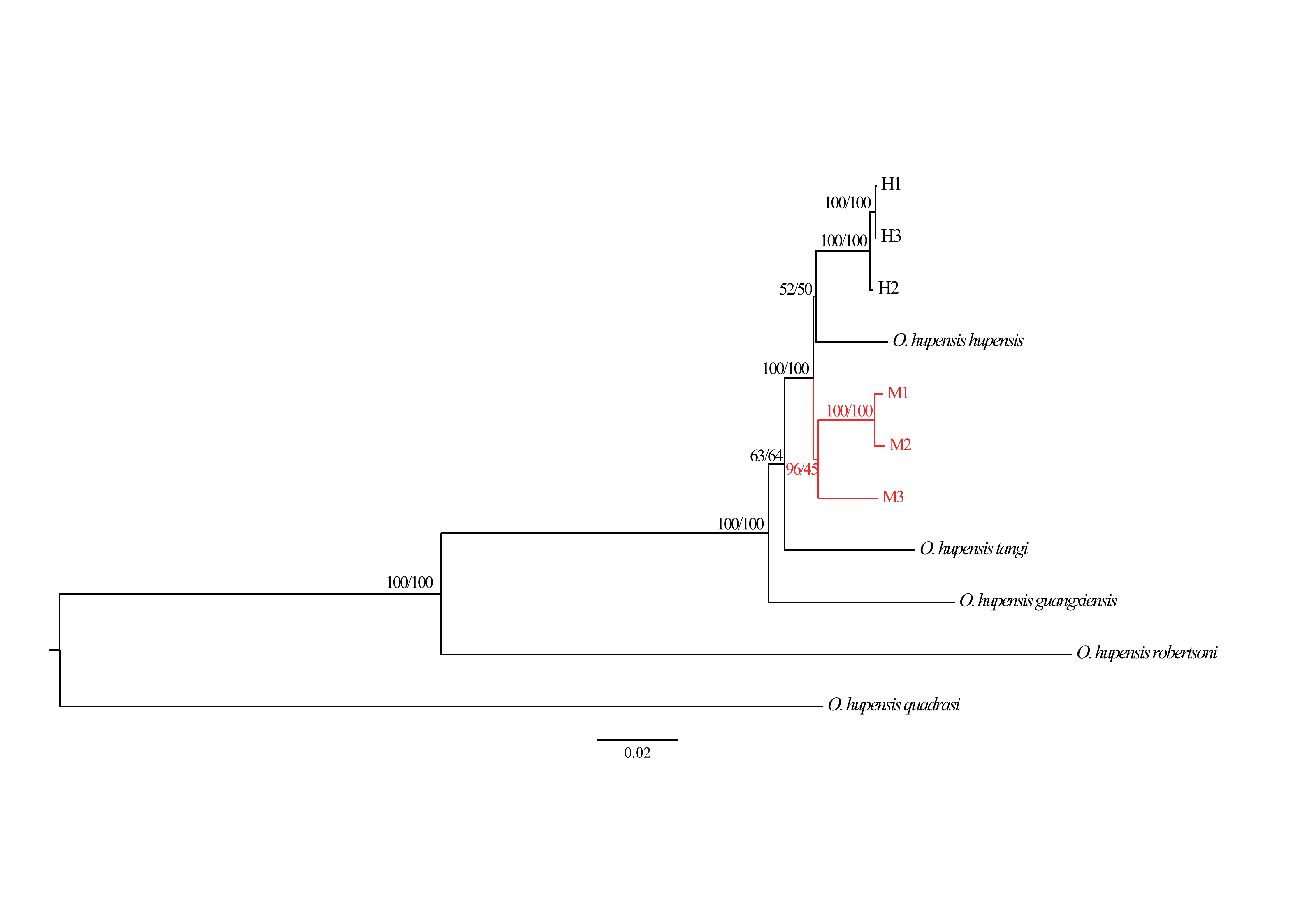


**Figure S1. The phylogenetic tree of five subspecies of *O. hupensis* based on thirteen mitochondrial protein-coding genes.** Nodal support values are the maximum-likelihood values/Bayesian posterior probabilities shown as percentages; H1-H3 indicate three individuals of hilly snails and M1-M3 represent three marshland snails.


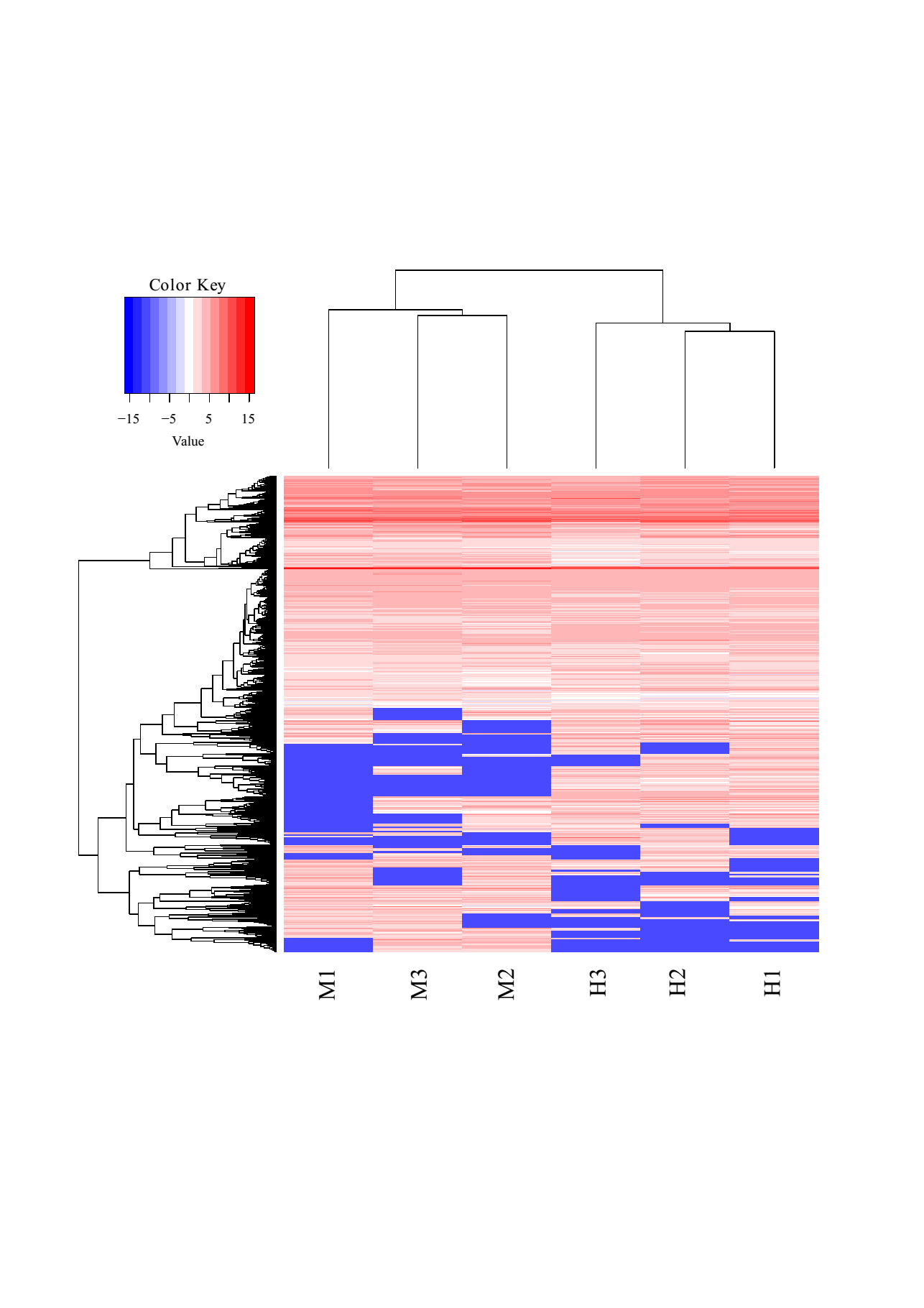


**Figure S2.** Global transcriptomic patterns of the six snails presented by hierarchical clustering of expression levels for all 34,760 unigenes.


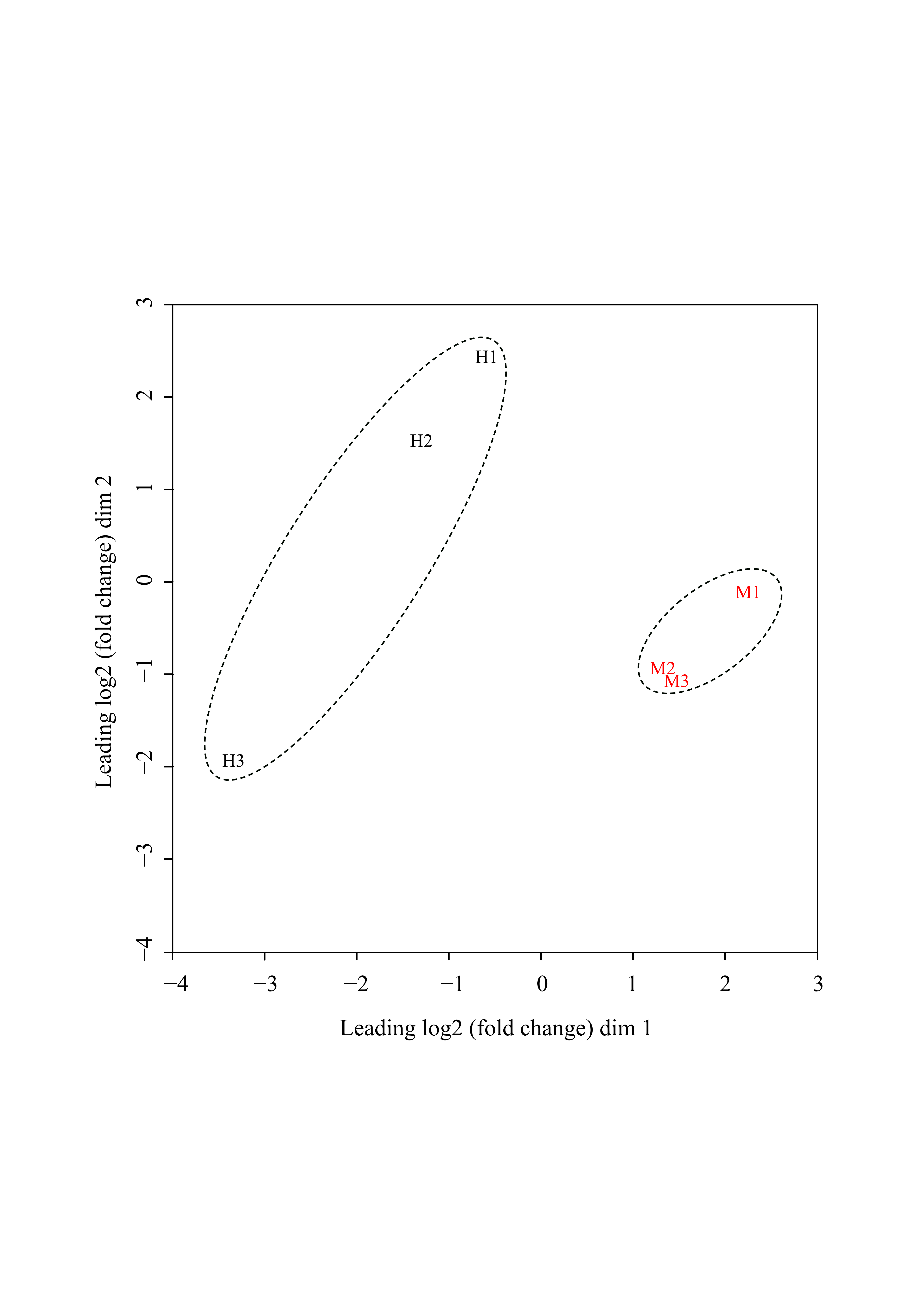


**Figure S3. The Multi-Dimension Scale (MDS) plot.** The plot was generated by the edgeR package based on read counts mapping to each contig.

**Table S1. GO enrichment of differential expressed genes in the marshland snails.**

| Category | GO id | Term | Count | *p*-value |
| --- | --- | --- | --- | --- |
| BP | GO:0006412 | translation | 17 | 8.53E-06 |
| BP | GO:0043043 | peptide biosynthetic process | 17 | 1.40E-05 |
| BP | GO:0043604 | amide biosynthetic process | 17 | 2.79E-05 |
| BP | GO:0006518 | peptide metabolic process | 17 | 1.10E-04 |
| BP | GO:0043603 | cellular amide metabolic process | 17 | 5.62E-04 |
| BP | GO:1901564 | organonitrogen compound metabolic process | 25 | 8.24E-04 |
| BP | GO:1901566 | organonitrogen compound biosynthetic process | 19 | 9.92E-04 |
| BP | GO:0042255 | ribosome assembly | 5 | 0.002411 |
| BP | GO:2001242 | regulation of intrinsic apoptotic signaling pathway | 5 | 0.006108 |
| BP | GO:0044036 | cell wall macromolecule metabolic process | 3 | 0.011365 |
| BP | GO:2001243 | negative regulation of intrinsic apoptotic signaling pathway | 4 | 0.012028 |
| BP | GO:0006457 | protein folding | 6 | 0.012235 |
| BP | GO:1903311 | regulation of mRNA metabolic process | 5 | 0.013884 |
| BP | GO:0007568 | aging | 6 | 0.014518 |
| BP | GO:0009628 | response to abiotic stimulus | 10 | 0.015292 |
| BP | GO:0042254 | ribosome biogenesis | 8 | 0.015424 |
| BP | GO:0044271 | cellular nitrogen compound biosynthetic process | 29 | 0.019526 |
| BP | GO:0040035 | hermaphrodite genitalia development | 3 | 0.020867 |
| BP | GO:1901576 | organic substance biosynthetic process | 36 | 0.022407 |
| BP | GO:0034645 | cellular macromolecule biosynthetic process | 29 | 0.024324 |
| BP | GO:0080135 | regulation of cellular response to stress | 7 | 0.025836 |
| BP | GO:0097193 | intrinsic apoptotic signaling pathway | 5 | 0.026137 |
| BP | GO:0005976 | polysaccharide metabolic process | 4 | 0.026599 |
| BP | GO:0000027 | ribosomal large subunit assembly | 3 | 0.029533 |
| BP | GO:0000272 | polysaccharide catabolic process | 3 | 0.029533 |
| BP | GO:0007567 | parturition | 2 | 0.030027 |
| BP | GO:0043484 | regulation of RNA splicing | 4 | 0.031688 |
| BP | GO:0042273 | ribosomal large subunit biogenesis | 4 | 0.031688 |
| BP | GO:0009059 | macromolecule biosynthetic process | 29 | 0.03205 |
| BP | GO:0009058 | biosynthetic process | 36 | 0.032347 |
| BP | GO:0005975 | carbohydrate metabolic process | 10 | 0.034371 |
| BP | GO:0022618 | ribonucleoprotein complex assembly | 6 | 0.035373 |
| BP | GO:0022613 | ribonucleoprotein complex biogenesis | 9 | 0.035933 |
| BP | GO:0048609 | multicellular organismal reproductive process | 8 | 0.036622 |
| BP | GO:0050684 | regulation of mRNA processing | 4 | 0.037251 |
| BP | GO:0032504 | multicellular organism reproduction | 8 | 0.039123 |
| BP | GO:0071826 | ribonucleoprotein complex subunit organization | 6 | 0.041029 |
| BP | GO:0071554 | cell wall organization or biogenesis | 3 | 0.042898 |
| BP | GO:0016998 | cell wall macromolecule catabolic process | 2 | 0.044705 |
| BP | GO:0006915 | apoptotic process | 12 | 0.045782 |
| BP | GO:1903409 | reactive oxygen species biosynthetic process | 3 | 0.046533 |
| BP | GO:0044238 | primary metabolic process | 55 | 0.046989 |
| CC | GO:0005840 | ribosome | 12 | 2.06E-05 |
| CC | GO:0022626 | cytosolic ribosome | 8 | 6.71E-05 |
| CC | GO:0044391 | ribosomal subunit | 9 | 2.92E-04 |
| CC | GO:0022625 | cytosolic large ribosomal subunit | 6 | 2.93E-04 |
| CC | GO:0005618 | cell wall | 4 | 0.001303 |
| CC | GO:0044445 | cytosolic part | 8 | 0.001569 |
| CC | GO:0030312 | external encapsulating structure | 4 | 0.001586 |
| CC | GO:1990904 | ribonucleoprotein complex | 16 | 0.002029 |
| CC | GO:0030529 | intracellular ribonucleoprotein complex | 16 | 0.002029 |
| CC | GO:0015934 | large ribosomal subunit | 6 | 0.004551 |
| CC | GO:0043232 | intracellular non-membrane-bounded organelle | 31 | 0.010176 |
| CC | GO:0043228 | non-membrane-bounded organelle | 31 | 0.010176 |
| MF | GO:0003735 | structural constituent of ribosome | 13 | 9.57E-07 |
| MF | GO:0019843 | rRNA binding | 7 | 7.52E-05 |
| MF | GO:0005198 | structural molecule activity | 14 | 1.09E-04 |
| MF | GO:0003723 | RNA binding | 23 | 9.12E-04 |
| MF | GO:0004553 | hydrolase activity, hydrolyzing O-glycosyl compounds | 6 | 0.002414 |
| MF | GO:0016798 | hydrolase activity, acting on glycosyl bonds | 6 | 0.004974 |
| MF | GO:0003746 | translation elongation factor activity | 3 | 0.034522 |
| MF | GO:0003676 | nucleic acid binding | 26 | 0.036157 |
| MF | GO:0000166 | nucleotide binding | 22 | 0.044997 |
| MF | GO:1901265 | nucleoside phosphate binding | 22 | 0.044997 |
| MF | GO:0055131 | C3HC4-type RING finger domain binding | 2 | 0.046038 |

**Table S2. GO enrichment of differential expressed genes in hilly snails.**

| Category | GO id | Term | Count | *p*-value |
| --- | --- | --- | --- | --- |
| BP | GO:0043043 | peptide biosynthetic process | 18 | 5.59E-06 |
| BP | GO:0043604 | amide biosynthetic process | 18 | 1.17E-05 |
| BP | GO:0006518 | peptide metabolic process | 19 | 1.33E-05 |
| BP | GO:0006412 | translation | 17 | 1.47E-05 |
| BP | GO:0043603 | cellular amide metabolic process | 20 | 2.55E-05 |
| BP | GO:0002181 | cytoplasmic translation | 7 | 4.18E-05 |
| BP | GO:1901566 | organonitrogen compound biosynthetic process | 21 | 2.08E-04 |
| BP | GO:0002376 | immune system process | 16 | 0.001795 |
| BP | GO:1901564 | organonitrogen compound metabolic process | 24 | 0.003566 |
| BP | GO:0008015 | blood circulation | 6 | 0.004349 |
| BP | GO:0003013 | circulatory system process | 6 | 0.004627 |
| BP | GO:0048514 | blood vessel morphogenesis | 7 | 0.005336 |
| BP | GO:0006955 | immune response | 10 | 0.005828 |
| BP | GO:0050880 | regulation of blood vessel size | 4 | 0.006729 |
| BP | GO:0002252 | immune effector process | 7 | 0.010216 |
| BP | GO:0008217 | regulation of blood pressure | 4 | 0.010221 |
| BP | GO:0003018 | vascular process in circulatory system | 4 | 0.010221 |
| BP | GO:0001568 | blood vessel development | 7 | 0.011049 |
| BP | GO:0001570 | vasculogenesis | 3 | 0.012279 |
| BP | GO:0003008 | system process | 11 | 0.01404 |
| BP | GO:0001944 | vasculature development | 7 | 0.014344 |
| BP | GO:0045087 | innate immune response | 7 | 0.014344 |
| BP | GO:0035150 | regulation of tube size | 4 | 0.014591 |
| BP | GO:0044271 | cellular nitrogen compound biosynthetic process | 30 | 0.019051 |
| BP | GO:0050886 | endocrine process | 3 | 0.019706 |
| BP | GO:0043542 | endothelial cell migration | 4 | 0.019871 |
| BP | GO:0002682 | regulation of immune system process | 9 | 0.024173 |
| BP | GO:0016525 | negative regulation of angiogenesis | 3 | 0.025465 |
| BP | GO:2000181 | negative regulation of blood vessel morphogenesis | 3 | 0.02857 |
| BP | GO:1901343 | negative regulation of vasculature development | 3 | 0.02857 |
| BP | GO:0001667 | ameboidal-type cell migration | 5 | 0.035113 |
| BP | GO:0006952 | defense response | 9 | 0.036046 |
| BP | GO:0044267 | cellular protein metabolic process | 32 | 0.036963 |
| BP | GO:0019538 | protein metabolic process | 34 | 0.044694 |
| BP | GO:0044236 | multicellular organism metabolic process | 3 | 0.046143 |
| BP | GO:0001525 | angiogenesis | 5 | 0.047394 |
| CC | GO:0022626 | cytosolic ribosome | 12 | 8.71E-09 |
| CC | GO:0044445 | cytosolic part | 13 | 2.32E-07 |
| CC | GO:0005840 | ribosome | 14 | 1.22E-06 |
| CC | GO:0044391 | ribosomal subunit | 12 | 1.86E-06 |
| CC | GO:0022627 | cytosolic small ribosomal subunit | 6 | 3.29E-05 |
| CC | GO:0022625 | cytosolic large ribosomal subunit | 6 | 4.16E-04 |
| CC | GO:0015935 | small ribosomal subunit | 6 | 5.24E-04 |
| CC | GO:0005912 | adherens junction | 10 | 1.50E-03 |
| CC | GO:1990904 | ribonucleoprotein complex | 17 | 1.63E-03 |
| CC | GO:0030529 | intracellular ribonucleoprotein complex | 17 | 1.63E-03 |
| CC | GO:0070161 | anchoring junction | 10 | 0.0017 |
| CC | GO:0005924 | cell-substrate adherens junction | 8 | 0.002085 |
| CC | GO:0005925 | focal adhesion | 8 | 0.002085 |
| CC | GO:0030055 | cell-substrate junction | 8 | 0.002307 |
| CC | GO:0015934 | large ribosomal subunit | 6 | 0.006262 |
| CC | GO:0030054 | cell junction | 12 | 0.023624 |
| CC | GO:0031225 | anchored component of membrane | 3 | 0.040772 |
| MF | GO:0003735 | structural constituent of ribosome | 13 | 1.30E-06 |
| MF | GO:0019843 | rRNA binding | 8 | 7.65E-06 |
| MF | GO:0005198 | structural molecule activity | 14 | 1.46E-04 |
| MF | GO:0004872 | receptor activity | 8 | 7.48E-03 |
| MF | GO:0060089 | molecular transducer activity | 8 | 7.48E-03 |
| MF | GO:0005488 | binding | 69 | 0.008283 |
| MF | GO:0008236 | serine-type peptidase activity | 5 | 0.008587 |
| MF | GO:0003729 | mRNA binding | 6 | 0.009162 |
| MF | GO:0017171 | serine hydrolase activity | 5 | 0.010483 |
| MF | GO:1901363 | heterocyclic compound binding | 40 | 0.020076 |
| MF | GO:0097159 | organic cyclic compound binding | 40 | 0.022656 |
| MF | GO:0004252 | serine-type endopeptidase activity | 4 | 0.025512 |
| MF | GO:0033218 | amide binding | 5 | 0.026294 |
| MF | GO:0003723 | RNA binding | 19 | 0.030875 |
| MF | GO:1990932 | 5.8S rRNA binding | 2 | 0.031781 |
| MF | GO:0004871 | signal transducer activity | 7 | 0.036785 |
| MF | GO:0003676 | nucleic acid binding | 26 | 0.049632 |

**Table S3. Primer sequences used in this study.**

| Genes | Forward primers (5’-3’) | Reverse primers (5’-3’) |
| --- | --- | --- |
| *O_actin** | TCGTGCGTGACATCAAGGAG | CGAGGAAGGAAGGCTGGAAA |
| *LRP5* | TCATCTGCAACATGGACAATAC | GACCACTGTATAACTGTACG |
| *P4HA2* | GCAACTATTCAGGTTATCTGG | GCCATTTGCAGCTTTCTTCAT |
| *BHMT* | CATGTTCAAGGAGCAGGTAC | CTAGGGTCACTACTGCTGGT |
| *PABP4* | GTTCCCGCAAGCTCAACGCT | CATTCCGCCACCGAGTTGAG |
| *FAP* | GGAGATGATAATGTCCACTTC | TCCAGACTGTTGAAAAGATG |
| *CEBPA* | TTCCAAGGGTGGGTCCAAGC | CGTCGTTAGCATCCAGCAGC |
| *AS3MT* | CGACCTTGTCATCTCCAATTG | TTCTCTGTCCGTGTACATGTC |
| *ATHL1* | GCTTGATGTATGGCAGAACC | CATGTAGGCCTGAGTGATG |
| *TEN1* | TGTACTAGTGCCAGCGTCTG | CGTTTGTCGCACCGGACATC |

* Primers used in this study were designed by Zhang et al. 2012. *Dev Comp Immunol* 36:241-246.
